# The Universities and the Nursing Profession

**Published:** 1920-03-27

**Authors:** 


### THE MATRONS' AND SISTERS' DEPARTMENT.

### THE UNIVERSITIES AND THE NURSING PROFESSION.

The London University has been the first to recognise the claims of nurses to special professional training. Through the broad organisation of King's College for Women the doors have been opened and a course has been arranged to meet the requirements of nurses who desire to study the things which belong to their calling more thoroughly than is possible during their hospital training.

The valuable pioneer work at King's ought not long to remain in splendid isolation. There are many provincial universities where the success of a course intended chiefly for nurses and women desirous of becoming nurses would be assured. In some university towns, Sheffield, for instance, the ground is being prepared by the institution of valuable post-graduate courses for nurses, under the initiative of the local Centre of the College of Nursing. At Liverpool the times are ripe for the University to have a hand in such a venture. At Manchester and Bristol there can be little doubt that once started a course for nurses would justify itself within a couple of years. The important matter is to get things under way for a start.

Why must the years between eighteen and twenty-one be tacitly ignored in this vital matter of the higher preparation of nurses for teaching and professional work? The nursing profession loses more than it can ever replace by the assumption that not until a woman has passed through her hospital career is she fitted to undergo any part of the special nursing course which will prepare her for the higher administrative posts in institutions and for municipal work. The truth is that a great deal of the training which nursing sisters take at King's College for Women in completion of their previous hospital course, is of a nature to be very readily imparted to girls fresh from the High School. Much, too, of the instruction indispensable for health visitors could be imparted at the same stage. It does not need any previous knowledge of nursing. It demands no more than an alert mind.

It is quite true that there is a particular kind of training for the post of matron and of sister-tutor which could not conceivably be given to any but trained nurses. The intensive course which has now been arranged for nursing sisters after the hospital certificate has been gained is of the utmost value to the individual and to the profession at large. Not for a moment would we seem to undervalue its introduction into the curriculum of such as have proved during their training their fitness for higher posts. But already the course begins to be found a trifle overloaded. Much which ought to have been mastered previously has to be included in it. It becomes evident that in the long years between leaving school and finally starting on a nursing career much time is wasted which might be turned to priceless account.

The great need of the moment is to catch those who are leaving school, and ground them in the things which will later on advance them in the career of nursing. A course extending over a year or eighteen months for extern pupils would, we believe, meet with immediate success not only in London but in all large centres. It would need very careful arrangement. But a wise director, herself a practised tutor in nursing, would be able to instil sound ideals and develop the practical studies on lines which would give no false smattering of nursing-lore, but would yet prepare the student for her future life as a nurse. Cooking, domestic economy, and the like would have a place in such a course. Business training would protect the pupils from pitfalls in divers directions. Physical training would enable them to meet hard work without flinching. And there would be special courses in subjects which all trained nurses cannot study later for lack of time and opportunity. It is during this preparatory period that massage or dispensing could be undertaken or the certificates necessary for the Health Visitor and Sanitary Inspector could be gained by women who desired to enter these callings. But all the time it would be kept steadfastly before the students that the natural crown of their career lay in nursing. To assume that the students at such a centre would rest content with the beginning and stop short of getting trained as nurses is to ignore the strong call of the profession to women with a vocation. The reforms now taking place in the conditions of training and future prospects of nurses will have a far-reaching effect before long. The need of the moment is to bring young women of eighteen into touch with nursing ideals as they leave school.

